# Association of body mass index with ER, PR and 14-3-3σ expression in tumor and stroma of type I and type II endometrial carcinoma

**DOI:** 10.18632/oncotarget.17209

**Published:** 2017-04-18

**Authors:** Joseph F. Peevey, Brandon-Luke L. Seagle, Kruti P. Maniar, J. Julie Kim

**Affiliations:** ^1^ Department of Pathology, Northwestern University Feinberg School of Medicine, Chicago, IL, USA; ^2^ Division of Gynecologic Oncology, Department of Obstetrics and Gynecology, Northwestern University Feinberg School of Medicine, Chicago, IL, USA; ^3^ Division of Reproductive Science in Medicine, Department of Obstetrics and Gynecology, Northwestern University Feinberg School of Medicine, Chicago, IL, USA

**Keywords:** endometrial cancer, PR, ER, 14-3-3, obesity

## Abstract

Obesity is a prominent risk factor for endometrial cancer (EC) and can impede on surgical and hormonal treatments. Markers of EC, estrogen receptor (ER), progesterone receptor (PR), phospho(Ser473)-AKT (pAKT) and 14-3-3 sigma (14-3-3σ) were measured in EC tissues in both the tumor and stroma and grouped by body mass index (BMI). Immunohistochemical scoring of 82 cases of Type 1 and Type II EC tissues revealed a significantly increased tumor expression of ER, PR and 14-3-3σ in women with Type I (BMI < 40) as compared to Type II (BMI < 30) EC. With higher BMI, only PR and 14-3-3σ in the tumor epithelium was significantly higher in Type I than Type II. In particular, Type I EC exhibited significantly increased levels of only PR from patients with BMI > 40 compared to BMI < 40. Type II EC showed increased expression of ER in the stroma only between high and low BMI. Analysis of the TCGA RNA-Seq mRNA expression of *ER, PR, PIK3CA, PTEN* and *SFN* (gene for 14-3-3σ) confirmed increased PR expression in EC of obese women. In conclusion, ER, PR and 14-3-3σ are differentially regulated in Type I compared to Type II EC while PR is dysregulated in obese women with Type I EC. These findings have potential implications for efficacy of progestin treatment in obese women.

## INTRODUCTION

Endometrial cancer (EC) is the most common gynecologic malignancy with an estimated 60,050 new cases and 10,470 deaths in the US in 2016 [1]. These numbers steadily increase each year. EC are traditionally classified as Type I or Type II based upon histology. Type I EC are most prevalent and exhibit frequent mutations in *PTEN*, members of the PI3K/AKT pathway, *FGFR2, ARID1A, CTNNB1, PIK3CA, PIK3R1 and KRAS* [2–4].

It remains unclear if tumor expression of estrogen receptor (ER) or progesterone receptor (PR) is independently associated with EC survival after adjusting for histologic type and tumor grade. While older studies suggested prognostic associations, a very recent analysis showed that knowing tumor ER or PR status did not improve prediction of survival [5, 6]. Moreover, ER and PR levels are traditionally assessed in the tumor cells exclusively and not in the surrounding stromal cells, despite expression of ER and PR in the stroma. Given the important paracrine actions of estrogen and progesterone between the epithelial/tumor and stromal cells [7–11], analysis of the stroma would provide additional and potentially useful information.

Prolonged unopposed estrogen exposure and conditions such as obesity, polycystic ovarian syndrome, estrogen-only hormone replacement therapy (HRT), and prolonged tamoxifen use, increase the risk of developing Type I EC [12–14]. The association of obesity and EC incidence is significant [15]. While frequently attributed to the mitogenic influence of excess estrogen, little is known about the mechanisms underlying this association. A BMI of 42 increases the risk of developing EC by ten-fold compared to normal weight [16]. Type II EC is less common and often presents at an advanced stage with poor prognosis leading to a disproportionate number of EC specific deaths. Type II EC include serous, clear cell, carcinosarcoma, and FIGO grade 3 endometrioid histologies, characterized by mutations in *TP53, CHD4*, and *FBXW7*, among others [17, 18].

We previously reported that resistance to progestins can occur due to hyperactivation of AKT signaling in endometrial tumor cells. We also discovered a novel partner of PR, 14-3-3σ, that is recruited with PR on chromatin and sensitizes EC cells to progestins [19]. 14-3-3σ proteins are ubiquitously expressed regulators of various cellular functions, including proliferation, metabolism, and differentiation [20]. 14-3-3σ is frequently decreased or lost in human cancers including breast, liver, prostate, lung and ovarian cancer [21–25].

The goal of this study was to determine the expression pattern of ER, PR, pAKT and 14-3-3σ in EC tissues from obese and non-obese women. Levels in both epithelial and stromal cells of Type I and Type II EC tumors were assessed and compared in relation to BMI.

## RESULTS

### Type I and Type 2 Endometrial cancer marker expression according to BMI

**Figure 1:**
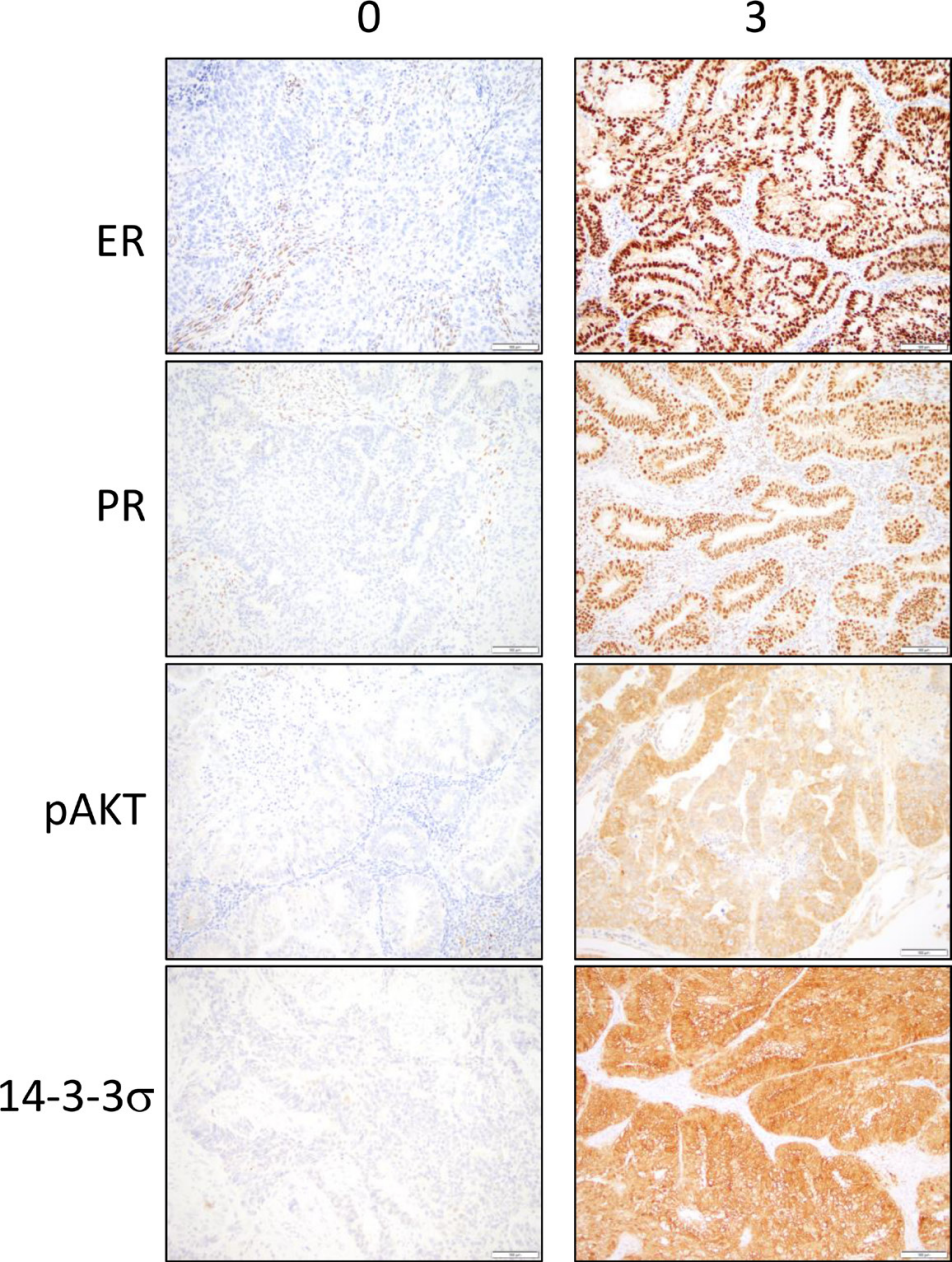
Immunohistochemical stainings for ER, PR, pAKT and 14-3-3σ in the tumor epithelium are observed in tumor cores from the endometrial cancer tissue microarray Representative sections of low expression (0) and high expression (3) are shown. Brown color represents positive staining.

**Figure 2:**
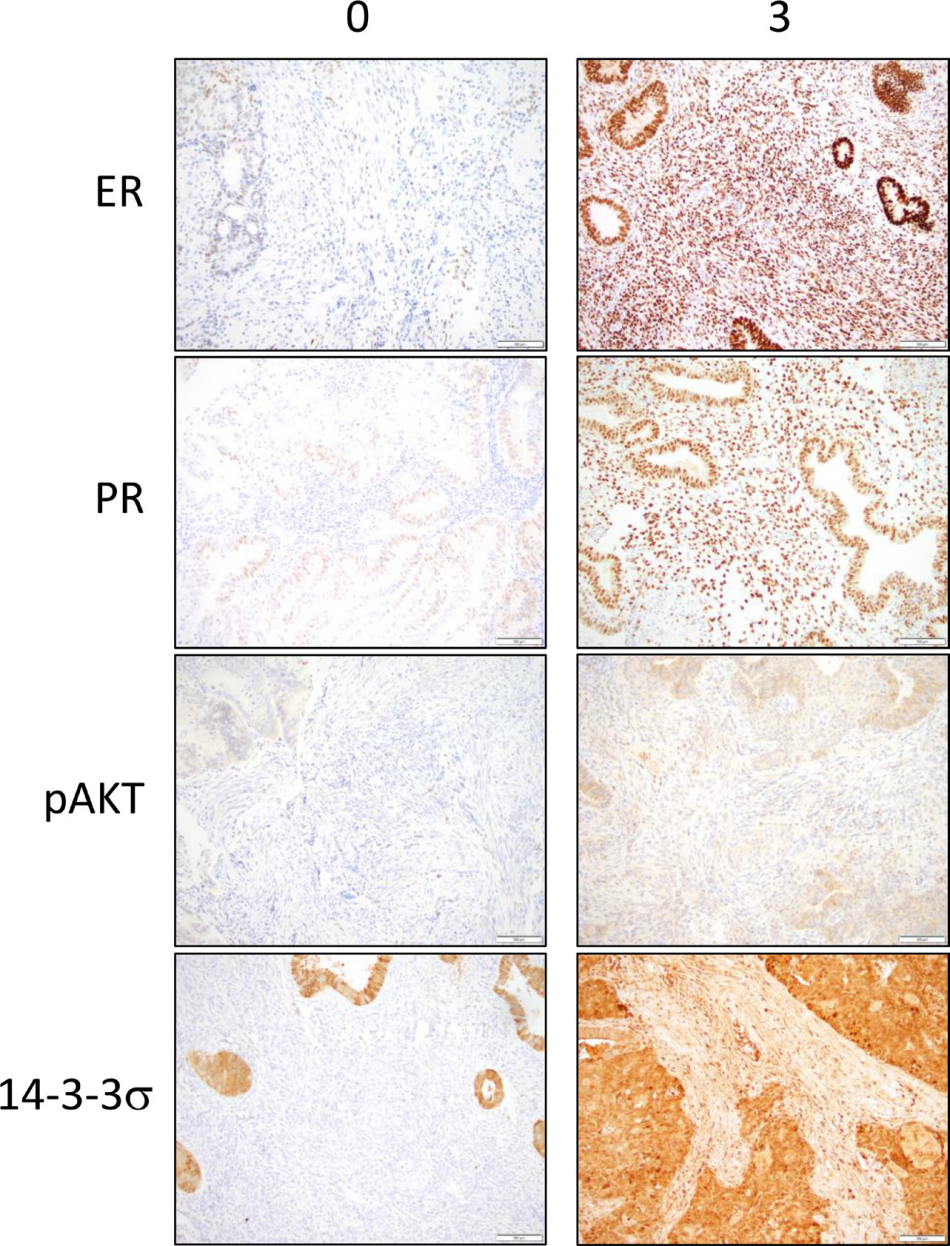
Immunohistochemical stainings for ER, PR, pAKT and 14-3-3σ in the stroma are observed in tumor cores from the endometrial cancer tissue microarray Representative sections of low expression (0) and high expression (3) are shown. Brown color represents positive staining.

**Figure 3:**
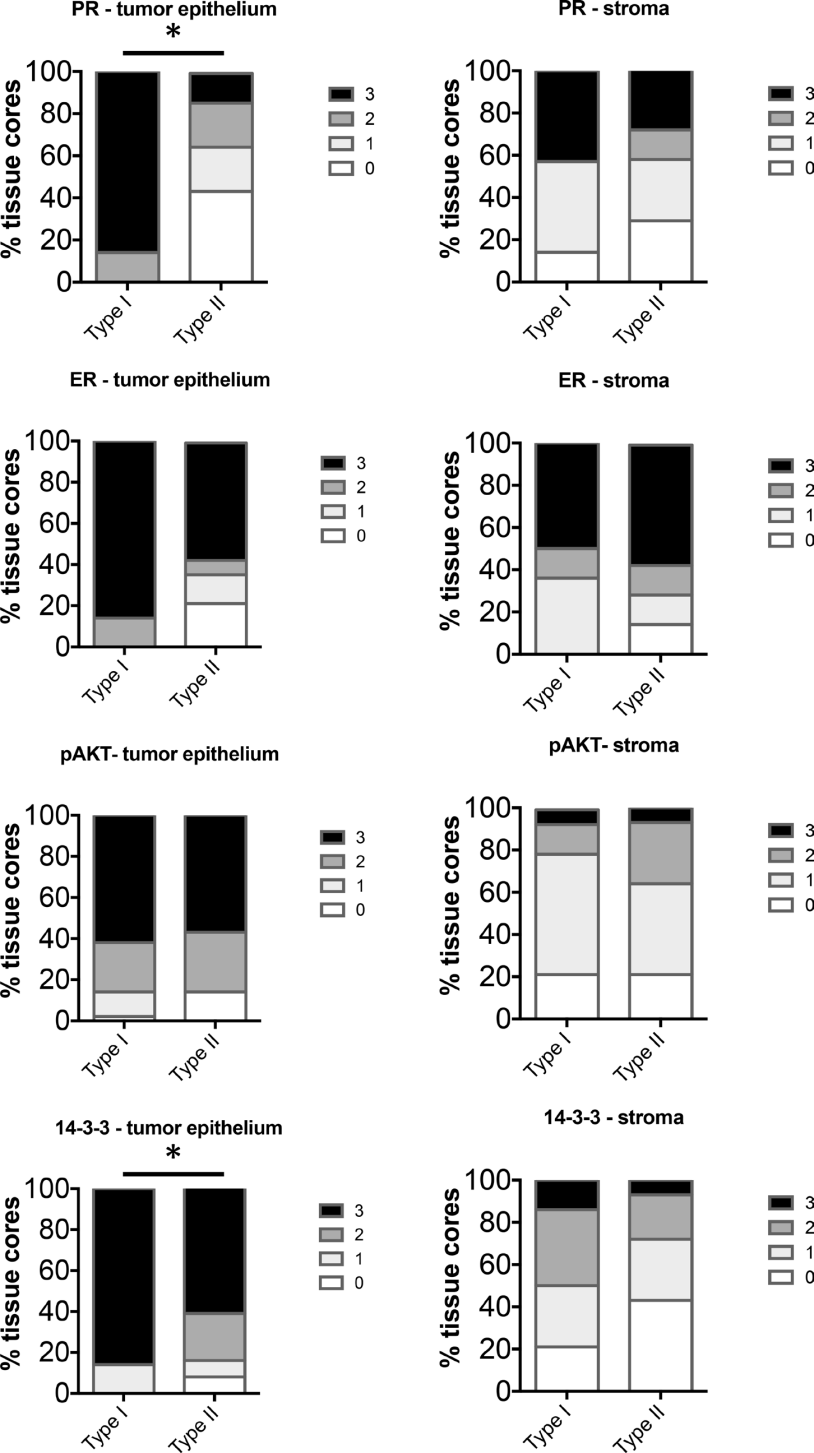
Comparison of distribution of intensity scores between Type I (BMI > 40) and Type II (BMI ≥ 30) tumors from obese women The intensity of staining in the tumor epithelium and stroma in each of the tissue cores was scored numerically as 0 (negative), 1 (weak), 2 (moderate), or 3 (strong). Statistically significant differences between Type I and Type II tissues were evaluated using the Mann-Whitney *U* test (*P < 0.05).

Clinical data including BMI and final pathological diagnosis were documented for the 82 cases represented on the tissue microarray (TMA). Cases were grouped by Type I (Grade 1 and 2 endometrioid adenocarcinoma, *n* = 56) and Type II (uterine serous, clear cell, carcinosarcoma, and grade 3 endometrioid adenocarcinoma, *n* = 26). In addition, these data were separated by BMI to group the data into obese and non-obese BMI. For Type I EC, cases were grouped by BMI > or < 40. For Type II, BMI > or < 30 was used due to the low number of cases with BMI of > 40. The frequency distribution of staining scores (0–3) was calculated and statistically analyzed. Representative images for marker staining with scores of 0 and 3 are shown in the tumor epithelium (Figure 1) and stroma (Figure 2). The percent frequency distribution was then plotted as contingency graphs. In obese cases, expression of PR and 14-3-3σ in the tumor epithelium was significantly higher in Type I compared to Type II EC (Figure 3). In Type I EC, PR levels in the tumor epithelium were scored at 2 (14%) or 3 (86%) as opposed to Type II tumors which exhibited lower PR levels (43% at 0, 21% at 1, 21% at 2, and 14% at 3). For 14-3-3σ, 86% of Type I stroma epithelium scored highly at 3 as opposed to 62% in Type II tumors. ER and pAKT levels did not differ statistically for frequency of score distribution between the two types of EC. In addition, no differences in staining were observed in the tumor stroma.

**Figure 4:**
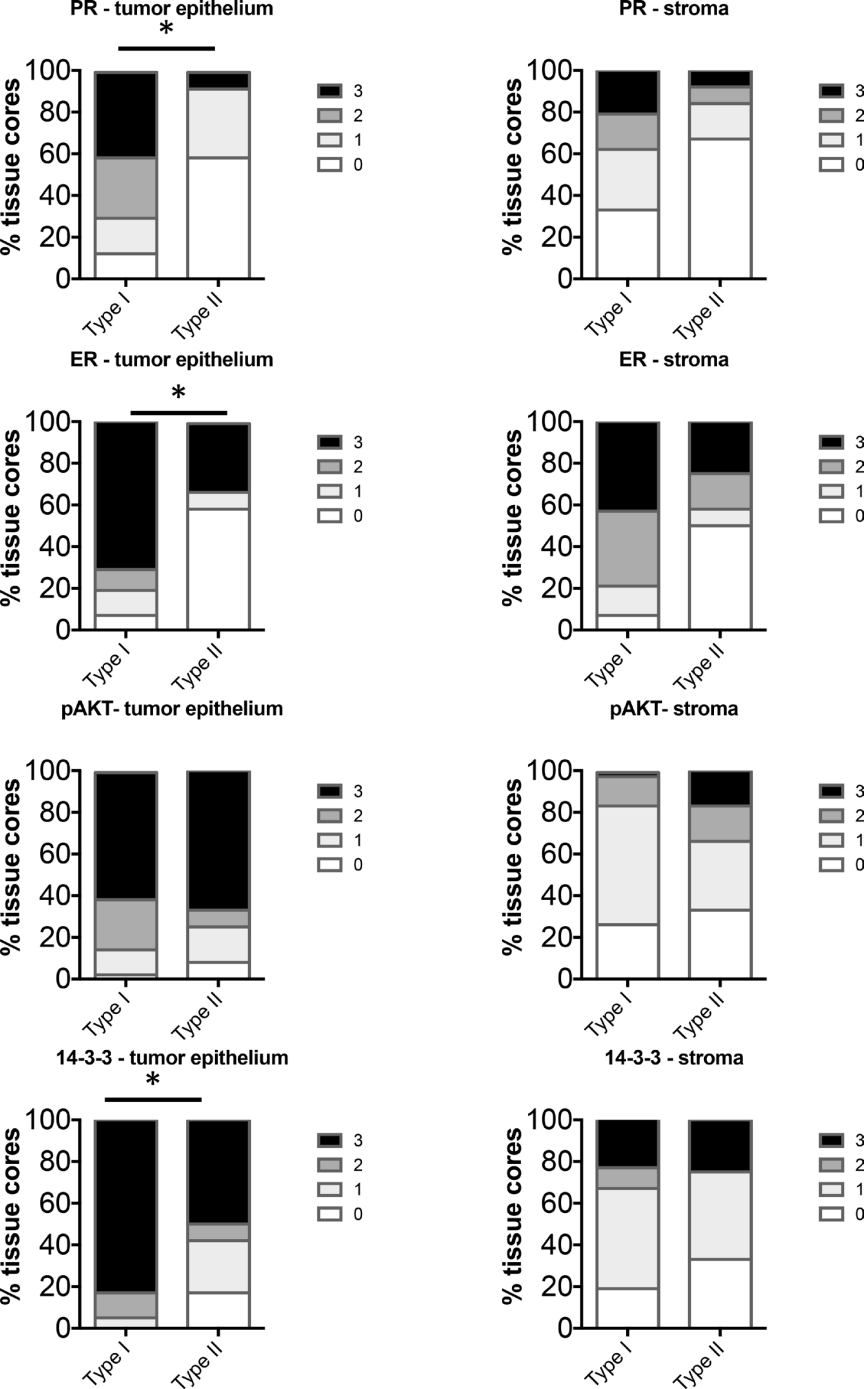
Comparison of distribution of intensity scores between Type I (BMI < 40) and Type II (BMI < 30) tumors from non-obese women The intensity of staining in the tumor epithelium and stroma in each of the tissue cores was scored numerically as 0 (negative), 1 (weak), 2 (moderate), or 3 (strong). Statistically significant differences between Type I and Type II tissues were evaluated using the Mann-Whitney *U* test (*P < 0.05).

Comparison of Type I and Type II EC in non-obese women (BMI < 40 or <30 respectively), revealed significant increase in levels of PR, ER and 14-3-3σ in the tumor epithelium in Type I EC (Figure 4). Levels of pAKT did not differ significantly in the tumor epithelium. In the stroma, despite positive staining, there was no significant difference in expression of PR, ER, pAKT or 14-3-3σ between Type I and Type II. Interestingly, in our cohort of cases, the Type I tumors with BMI between 40 and 30 exhibited staining levels similar to cases of BMI < 30 (data not shown) and thus these cases were combined in one group (BMI < 40).

### Endometrial cancer marker expression in Type I tumors comparing obese and non-obese cases

**Figure 5:**
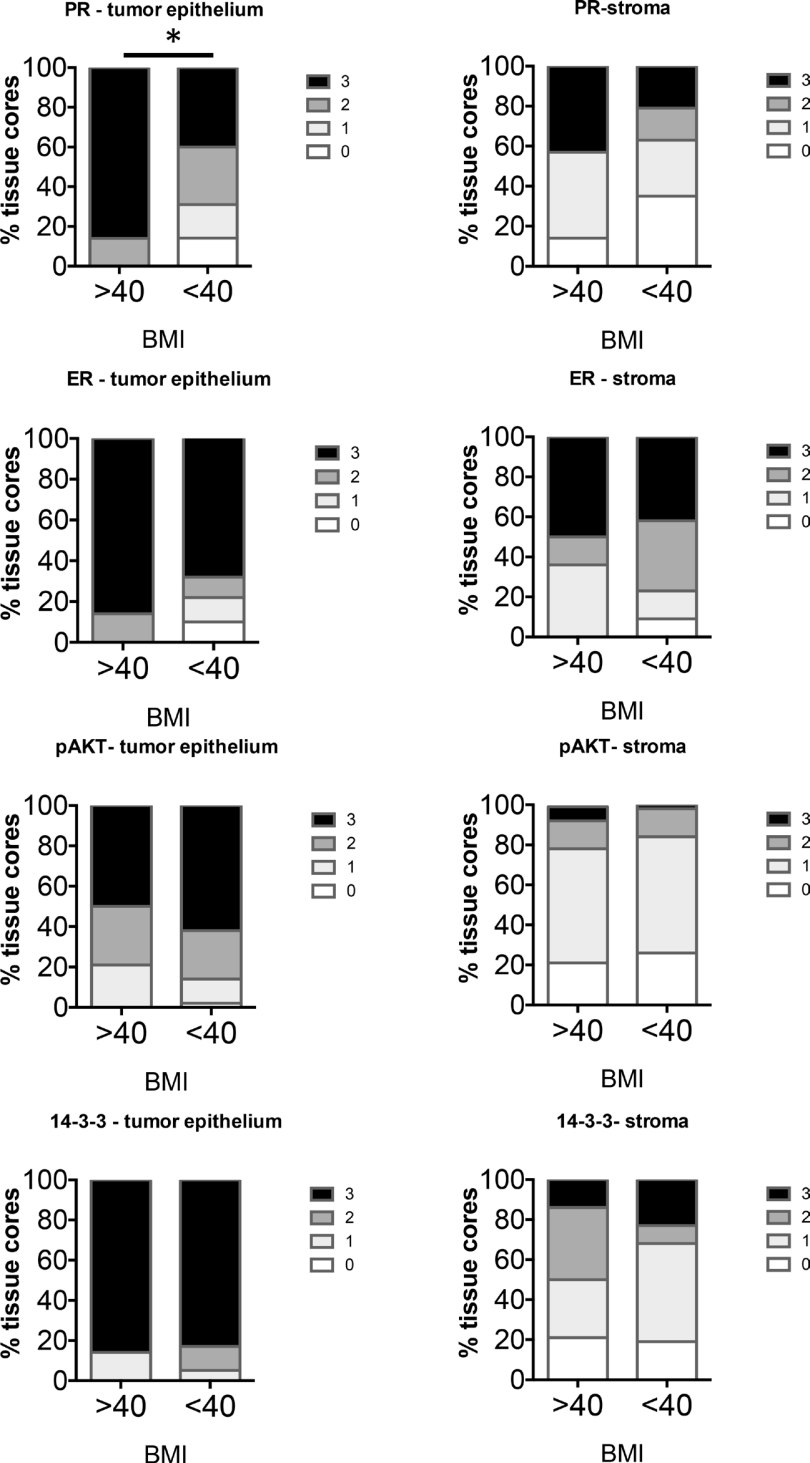
Comparison of distribution of intensity scores of Type I tumors from women with BMI > or < 40 The intensity of staining in the tumor epithelium and stroma in each of the tissue cores was scored numerically as 0 (negative), 1 (weak), 2 (moderate), or 3 (strong). Statistically significant differences between tissues from BMI > or <40 were evaluated using the Mann-Whitney *U* test (*P < 0.05).

Levels of markers were compared in Type I EC in BMI > vs < 40. There were a total of 14 Type I cases of BMI > 40 and 43 cases of BMI < 40. Analysis of the percent frequency distribution of scores showed a significant difference in only the PR levels in the tumor epithelium while other markers in the tumor epithelium as well as the stroma did not show significant differences in staining (Figure 5). PR in the tumors of BMI > 40 cases scored higher than BMI < 40 cases.

**Figure 6:**
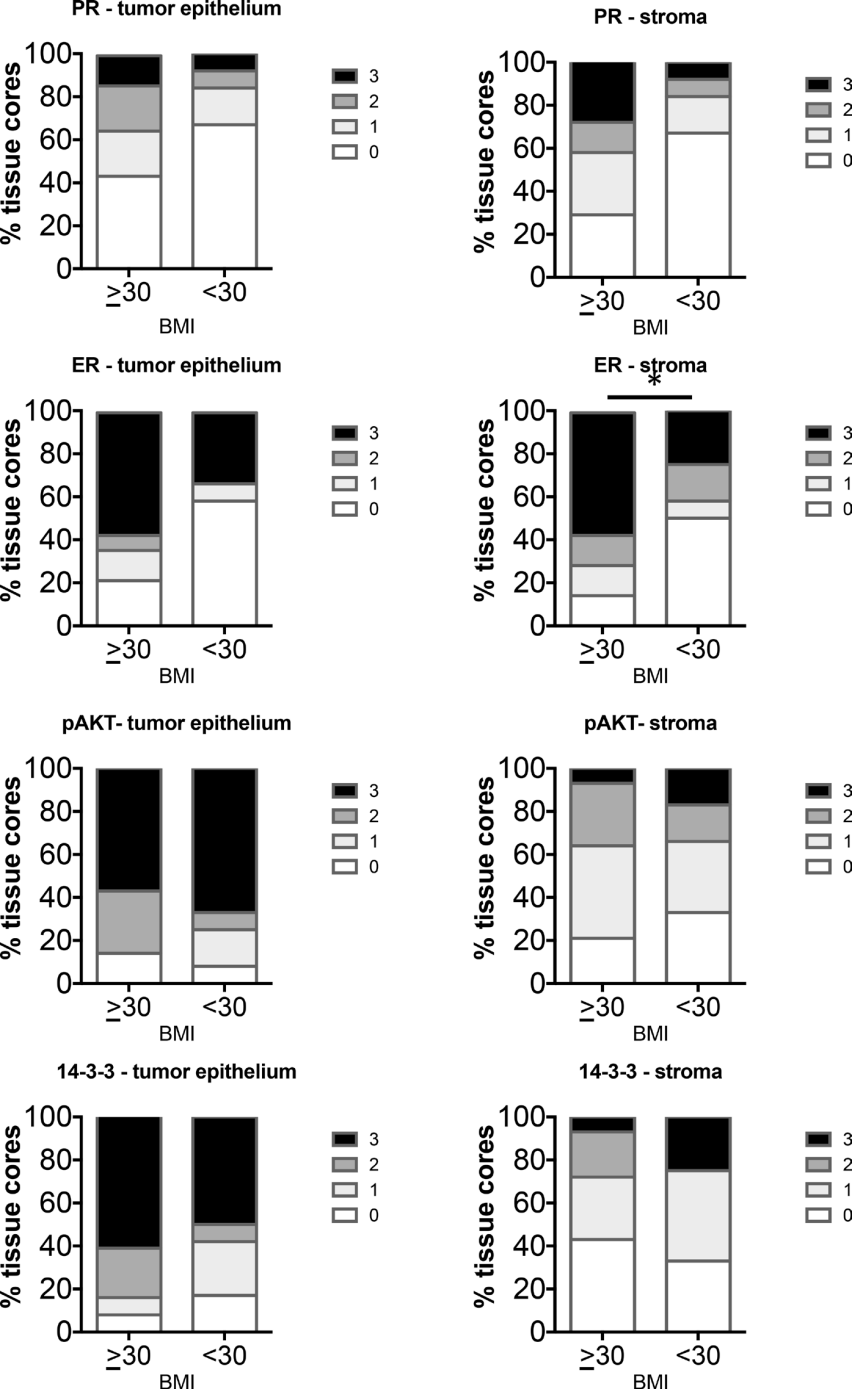
Comparison of distribution of intensity scores of Type II tumors from women with BMI ≥ or < 30 The intensity of staining in the tumor epithelium and stroma in each of the tissue cores was scored numerically as 0 (negative), 1 (weak), 2 (moderate), or 3 (strong). Statistically significant differences between tissues from BMI ≥ or < 30 were evaluated using the Mann-Whitney *U* test (*P < 0.05).

Type II tumors were grouped as BMI > or < 30 as only 1 case was from a woman with a BMI > 40. In total, 14 Type II cases of BMI > 30 and 12 cases BMI < 30 were analyzed. The group with BMI > 30 exhibited increased staining of ER in the stroma compared to non-obese women (Figure 6). None of the other markers were significantly different in the tumor epithelium or stroma in the two groups.

### Endometrial cancer marker expression from the cancer genome atlas (TCGA) by BMI

In order to determine whether the EC markers studied here differed in obese and non-obese women at the mRNA level, we explored the existing TCGA database that was generated in endometrial carcinoma [3]. We did a preliminary query of 6 genes that were of interest to us (Table 1) and grouped the transcriptomic data by BMI [morbidly obese (BMI > 40) or non-obese (BMI < 30)] for only endometrioid cases (Type 1). There were 37 cases of non-obese women and 24 women of morbidly obese women available that fit these criteria. Preliminary analysis of 6 genes showed an increase of PR expression in morbidly obese women by 4 fold. ESR1, ESR2, and PIK3CA expression were not significantly different, PTEN was slightly elevated (1.25 fold, *p* = 0.04) and interestingly the gene for 14-3-3σ, SFN was decreased (0.526, *p* = 0.054) (Table 1). Correlation tests showed analogous directional relationships of tumor PGR, PTEN and SFN mRNA expression across the range of observed BMI values (Table 2).

**Table 1:** Differential mRNA expression between primary tumors from women with BMI ≥ 40 (morbidly obese) versus BMI ≤ 30 (non-obese) from TCGA database

Gene	Exp - BMI < 30 (*n* = 37)	Exp - BMI > 40 (*n* = 24)	Fold Change	*P*
ESR1	5661 (2097–9880)	7317 (2895–11125)	1.29	0.775
ESR2	13 (8–20)	14 (10–21)	1.09	0.543
PGR	989 (231–3030)	4469 (918–8179)	4.52	0.055
PIK3CA	251 (195–354)	224 (160–336)	0.894	0.294
PTEN	1304 (916–1624)	1632 (1381–1993)	1.25	0.043
SFN	3170 (1877–5652)	1668 (1230–4546)	0.526	0.054

Exp is mRNA expression from primary tumor RNA Sequencing and is reported as median (IQR). P is from Mann-Whitney *U* test.

**Table 2:** Correlation test as BMI and mRNA expression are continuous variables

Gene	Spearman's rho	*P*
ESR1	0.051	0.623
ESR2	0.121	0.246
PGR	0.240	0.020
PIK3CA	−0.164	0.114
PTEN	0.204	0.048
SFN	−0.177	0.088

## DISCUSSION

This study revealed that among the EC markers studied, PR and 14-3-3σ protein levels were significantly higher in Type I versus Type II tumors, while only PR protein level expression was higher in Type I tumors when comparing tumors from women of BMI > 40 or BMI < 40. Analysis of the TCGA database confirmed that PR mRNA was higher in tumors from cases of BMI > 40 compared to BMI < 30. Other markers, ER and 14-3-3σ were significantly different only when comparing Type I versus Type II EC from patients with BMI < 40 or < 30 respectively. The differential regulation of PR between Type I and Type II cancers as well as within Type I cancers depending on BMI, supports the influential role of PR in this cancer. Recently, a group analyzed the TCGA database for EC to evaluate the differences in gene expression of obese and non-obese women with EC [26]. They identified 181 genes that were significantly up- or down-regulated with increasing BMI and among the genes PR expression was increased. Our data support the upregulation of PR both at the mRNA and protein levels. We were also able to stratify the expression of markers depending on BMI in each Type of EC as well as distinguish staining in the tumor epithelium and stroma. Of note was the significant increase in ER staining in the stroma of Type II EC in women of BMI > 30. While Type II cancers have not been considered as hormone-dependent, the role of hormones cannot entirely be ruled out. Analysis of sex hormone levels including estradiol, progesterone, testosterone, FSH and LH as well as levels of ER and PR in the tumors were measured in 187 women with EC [27]. Hormone levels were similar between the subtypes of EC regardless of menopausal status, and most of the Type I and II ECs were positive for ER and PR. Thus, the potential regulation of stromal ER in Type II EC in obese women is of interest and may implicate effects of the inflammatory microenvironment caused by obesity.

Studies show that up to 50% of EC cases treated with progestins fail to show a complete response [28]. Additionally, with advancing disease, the sensitivity to progestins decreases [29, 30]. Methylation of the PR promoter has been demonstrated in EC [31] resulting in decreased levels of PR. EC cells that were negative for PR can gain PR expression using epigenetic modulators [32]. Post-translational modifications of PR including phosphorylation, sumoylation, ubiquitination and acetylation can alter protein stability, localization and function [33]. Hyperactivated signaling pathways can promote post-translational modifications of PR as well as regulate important coregulators that influence PR action at the chromatin [19, 34]. Recently, we reported that hyperactivated AKT pathway in EC alters PR action and significantly affects the proteins that co-precipitate with PR at the chromatin [19]. We identified the 14-3-3 family of proteins to be recruited with PR upon inhibition of AKT and specifically, 14-3-3σ increased PR activity on a subset of genes. The higher expression of 14-3-3σ in Type I EC regardless of BMI compared to Type II cases suggests that 14-3-3σ could influence PR action preferentially in Type I.

Response rates to progestins for early EC vary among studies, which are usually limited to small case numbers. Upon review of published studies, response rates to progestins range from approximately 40% to 85% [28, 35–40]. Two studies reported that obese patients have lower response rates to progestins than non-obese patients [35, 41]. The higher PR expression observed in Type I EC with BMI > 40 in our study seemed counterintuitive to this, as higher levels of receptor are usually associated with increased response to the hormone. However, we know from our studies and others that there are numerous factors which influence PR transcriptional function and thus, it would be necessary to study this in the obese setting. An increase in hormone receptor levels could also be indicative of inactive receptors since active receptors are usually ubiquitinated and turned over rapidly [42, 43]. Despite the lack of statistical difference in 14-3-3σ at the protein level, the TCGA data showed lower SFN as well as higher PR expression in cases of BMI > 40, compared to BMI < 30. If indeed 14-3-3σ serves as an important regulator of PR transcriptional function in primary human tumors as we see for EC cell lines [19], this gene could be used in addition to PR to predict PR activity. Although earlier studies have associated ER and PR with a favorable response to progestins, PR positive tumors do not always respond [29]. Moreover, some ER/PR-negative tumors have exhibited response to progestins. Reasons for this are numerous [44] and require an in depth analysis especially in the context of obesity. Obesity is considered to be a disease of inflammation [45] and could influence PR action at the level of post-translational modifications, protein stability, localization and recruitment of essential coregulators, as well as its microenvironment. Additional studies are warranted to determine the influence of obesity on progestin response for EC.

Obesity has become a major health concern in the United States with 1 in 3 adults considered to be obese and about one-third of children and adolescents ages 6 to 19 to be overweight or obese [46, 47]. One study reported that obesity at a younger age increases risk for EC even if weight is lost in adulthood [48]. The prevalence of obesity and the rise in incidence of EC warrants better measures for prevention as well as early treatment. Our data raise consideration that background stromal expression of hormone receptors and their associated markers including 14-3-3σ may be considered in the development of predictive models of response to hormone treatment. Additionally, patient BMI should be investigated as a confounder or effect modifier of tumor epithelial and stromal marker expression as a predictor of response to hormonal treatment.

## MATERIALS AND METHODS

### Human endometrial cancer tissues

Uterine tumors were obtained from women who provided written informed consent prior to surgery. This study was approved by the Institutional Review Board of Northwestern University.

### Tissue microarray

A TMA was created by the Human Pathology Core at the Robert H Lurie Comprehensive Cancer Center at Northwestern University. The TMA consisted of a 7 × 14 grid of 1.5 mm tissue cores. The grid included 89 randomly positioned EC tissues as well as 4 control tissues of normal endometrium. Of the 89 tumors, 7 cases were eliminated from analyses as 2 cases were leiomyosarcoma and 5 were atypical hyperplasia.

### Immunohistochemistry

Immunohistochemistry was performed for the following proteins: ER (Clone SP1, Thermo Scientific Catalog # RM-91), PR (Clone PgR 636, Dako Catalog # M3569), p(Ser473)-AKT (Cell Signaling, Catalog # 12694), and 14-3-3σ (Bethyl Laboratories Catalog # A301 -648A). All Immunohistochemistry was performed at the Pathology Core Facility of the Robert H. Lurie Comprehensive Cancer Center of Northwestern University. Antibodies were tested on negative and positive control tissues provided by the Core Facility. Semiquantitative immunoreactivity for all markers was scored by a pathologist. Immunostains were scored as the percent of positively stained cells: [(0) absent; (1) < 10%; (2) 11–50%; (3) > 50%].

### The cancer genome atlas primary tumor mRNA expression by body mass index

Publicly available EC clinical and primary tumor mRNA gene expression datasets from TCGA were downloaded from cBioPortal [3, 49, 50]. Public data use for research was per TCGA policies [51]. Only tumors with endometrioid histology were analyzed. A total of 94 endometrioid tumors had data for mRNA expression and BMI. Primary tumor mRNA expression was compared between non-obese (BMI < 30 kg/m^2^, *n* = 37) and morbidly obese (BMI ≥ 40 kg/m^2^, *n* = 24) women using the Mann-Whitney U test. The correlation of mRNA expression and BMI was tested with Spearman's rank correlation test.

### Statistical analyses

The immunostaining score frequency distribution of TMAs was compared with the Mann-Whitney *U* test. Tumor mRNA expression from the TCGA endometrioid EC cohort were compared using the Mann-Whitney *U* test. Groupings for comparisons are indicated in Results. Statistical tests were performed with R version 3.3.1 (2016-06-21) [52].

## Abbreviations

14-3-3σ: 14-3-3 sigma
ARID1A: AT-rich interaction domain 1A
BMI: body mass index
CHD4: chromodomain helicase DNA binding protein 4
CTNNB1: catenin beta 1
EC: endometrial cancer
ER: estrogen receptor
FBXW7: F-box and WD repeat domain containing 7
FGFR2: fibroblast growth factor receptor 2
FSH: follicle stimulating hormone
KRAS: KRAS proto-oncogene, GTPase
LH: luteinizing hormone
pAKT: phospho(Ser473)-AKT
PIK3CA: phosphatidylinositol-4,5-bisphosphate 3-kinase catalytic subunit alpha
PIK3R1: phosphoinositide-3-kinase regulatory subunit 1
PR: progesterone receptor
PTEN: phosphatase and tensin homolog
SFN: stratifin (gene for 14-3-3σ)
TCGA: The Cancer Genome Atlas
TMA: Tissue microarray
TP53: tumor protein p53
